# Integration of ZnO and CuO nanowires into a thermoelectric module

**DOI:** 10.3762/bjnano.5.106

**Published:** 2014-06-30

**Authors:** Dario Zappa, Simone Dalola, Guido Faglia, Elisabetta Comini, Matteo Ferroni, Caterina Soldano, Vittorio Ferrari, Giorgio Sberveglieri

**Affiliations:** 1 SENSOR Lab, Dipartimento di Ingegneria dell’Informazione, University of Brescia, Via Valotti 9, 25133 Brescia, Italy; 2 CNR-INO, U.O.S. Brescia, SENSOR Lab, via Branze 45, 25123, Brescia, Italy; 3 Dipartimento di Ingegneria dell’Informazione, University of Brescia, via Branze 38, 25123, Brescia, Italy; 4 Currently at ETCs.r.l., via Gobetti 101, 40129 Bologna, Italy

**Keywords:** copper oxide, nanowires, thermoelectric, zinc oxide

## Abstract

Zinc oxide (ZnO, *n*-type) and copper oxide (CuO, *p*-type) nanowires have been synthesized and preliminarily investigated as innovative materials for the fabrication of a proof-of-concept thermoelectric device. The Seebeck coefficients, electrical conductivity and thermoelectric power factors (TPF) of both semiconductor materials have been determined independently using a custom experimental set-up, leading to results in agreement with available literature with potential improvement. Combining bundles of ZnO and CuO nanowires in a series of five thermocouples on alumina leads to a macroscopic prototype of a planar thermoelectric generator (TEG) unit. This demonstrates the possibility of further integration of metal oxide nanostructures into efficient thermoelectric devices.

## Introduction

A thermoelectric generator (TEG) is a device capable of converting a temperature gradient into an electrical voltage difference, and vice versa. Recent studies [1–2] demonstrate that improvements in the technology platform of thermoelectrics could make solar thermoelectric generators competitive with other solar power conversion methods such as photovoltaics or thermal systems. This could potentially boost efforts towards low-cost environmentally-friendly materials for high-temperature thermoelectrics, thus allowing new opportunities in the conversion of electrical energy from naturally-available ambient sources, as proposed by Kraemer et al. [2].

There are a number of factors that influence the thermoelectric (TE) performance of a material, including the thermal conductivity κ, the electrical conductivity σ and the Seebeck coefficient *S*. Further, the efficiency of a thermoelectric device depends on the thermoelectric power factor (TPF) and the figure of merit (ZT) of the material, which are defined as *S^2^*σ and *S*^2^*T*σ/κ, respectively (being *T* the temperature). State-of-the-art bulk materials-based thermoelectric generators have been demonstrated to possibly reach energy conversion efficiency values of 5.2% and higher, with materials characterized by a ZT factor approaching a unitary value [3–4].

Transition metal oxides (TMOs) are a family of thermoelectric material that are not widely explored in literature, even if well known in other research areas [5]. Their electronic and thermal properties can be controlled by tuning morphology, doping and stoichiometry, and thus are promising materials for the implementation of thermoelectric devices. In particular, metal oxide nanowires are low-dimensional structures showing very promising and exciting properties. As a result of their large degree of crystallinity, they exhibit excellent thermal stability at high temperature and good electrical conductivity, leading to enhanced TPF and thus enabling the development of high-efficiency and high-temperature thermoelectric devices [6–9].

Thermoelectric devices based on different types of nanowires (Bi_2_Te_3_ [10], Si [11–13]) have been previously proposed, exhibiting promising increases in performance. In this work, we present the fabrication by simple and low-cost physical methods of both *n*- and *p*-metal oxide nanowires in order to build a prototype of planar thermoelectric unit based on metal oxide nanowire arrays, targeting different applications ranging from radioisotope thermoelectric generators [14–16] to automotive industry with fuel economy improvements, and more [16–18]. Further, these modules can have a significant impact on energy harvesting applications for powering low-power portable electronics and autonomous sensor systems [19–21], which intrinsically must be independent of durability of the power supply.

A thermoelectric device mainly consists of a series of elementary thermoelectric units, which in the simplest assembly consist of a pair of *p* and *n* materials. A prototype of thermoelectric module has been fabricated [22], combining five *n*-(ZnO) and *p*-type (CuO) nanowires-based thermocouples electrically connected in series and thermally in parallel, and its thermoelectric properties have been measured.

## Results and Discussion

In order to build the module, first ZnO (*n*-type) and CuO (*p*-type) nanowires have been fabricated independently in order to evaluate their intrinsic and individual thermoelectric properties. ZnO nanowires were fabricated by physical vapour deposition (PVD) technique in a tubular furnace [23], while CuO nanowires were synthesized by thermal oxidation [24] (see Experimental for further details).

**Figure 1:**
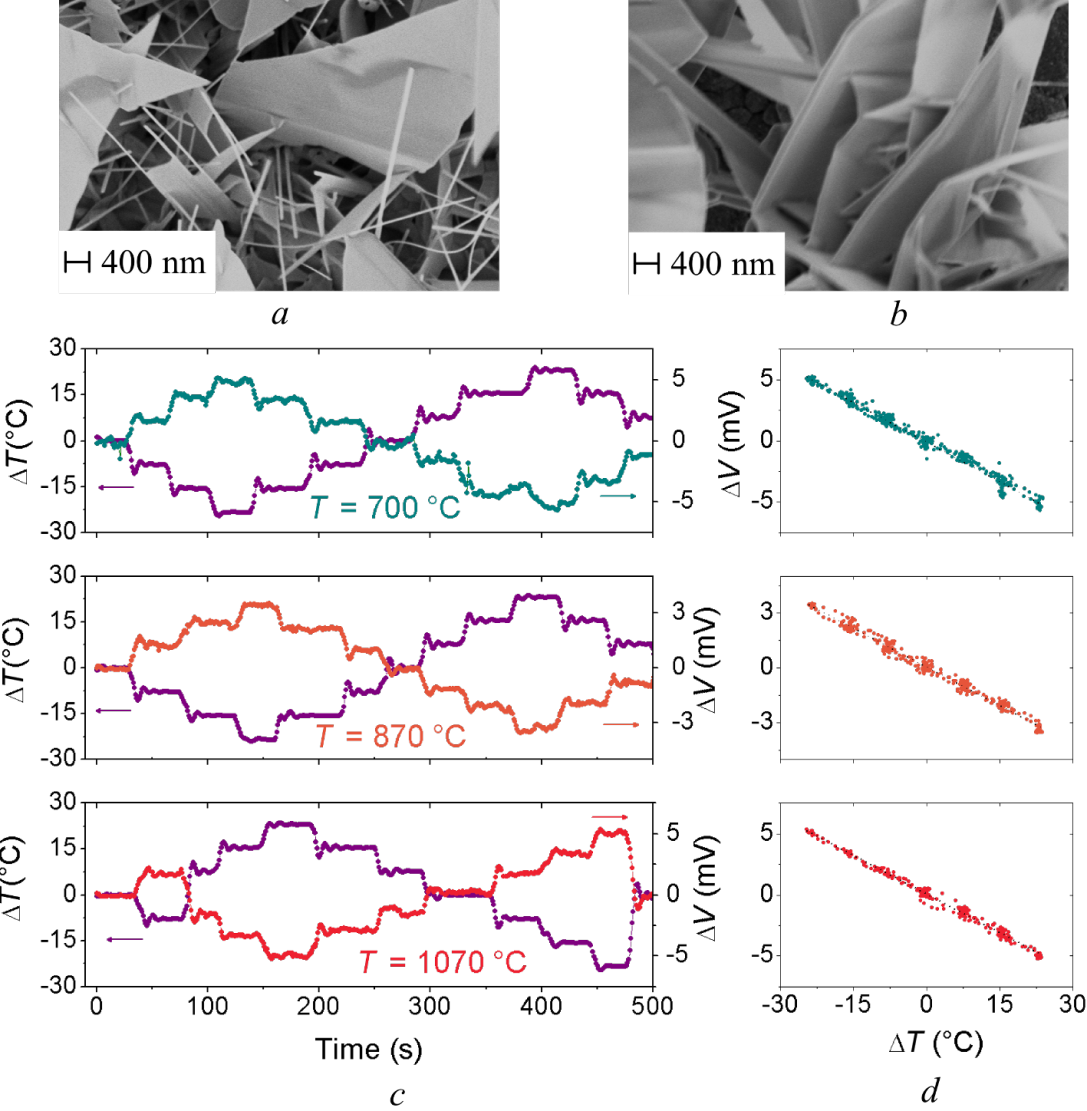
SEM images of ZnO nanowires deposited at substrate temperatures of (a) 700 °C and (b) 1070 °C. (c) Dependence of the applied temperature difference Δ*T* and the thermoelectric voltage Δ*V* as function of time and (d) voltage Δ*V* as function of the applied temperature difference Δ*T* across the ZnO nanowires samples [23].

Figure 1a and Figure 1b show the scanning electron microscopy (SEM) images of ZnO nanostructures fabricated by PVD technique [23]. Growth temperature has a strong influence on the nanostructures morphology; in fact, samples prepared at the highest temperature (1070 °C) show larger-size nanostructures as compared to low-temperature grown samples (700 °C). Further, the morphology of the latter appears more regular and uniform over the entire substrate. In this perspective, we should consider that Seebeck coefficient, thermal and electrical conductivity could vary depending on the nanowires direction respect to the longitudinal axis. However, it should be considered that due to the very small diameter of the nanowires (compared to the length) the thermal gradient induced along this direction can be neglected. Further, being the nanowires in a mat configuration (both for ZnO and CuO), we can additionally assume that the dominant contribution to the electrical conductance comes from the electrical conductivity along the axis of the nanowires. As-grown nanowires do not show any particular alignment respect to the substrate surface, as a result of combined growth mechanism and mechanical stress. Some degree of vertical alignment would not have improved the overall properties, but worsen instead since the thermal gradient would have then been applied horizontally. In addition, there is no matrix surrounding the mats, being self-supported in atmosphere.

Nanowires are connected and form semiconductor-to-semiconductor junctions; energy barriers form at these locations, with the overall effect of increasing the total electrical resistance of the device. A reduction in the nanowire length will increase the number of junctions that charge carriers have to face within the entire extension of the mat, thus increasing the total electrical resistance and reducing the efficiency. Instead, the presence of wall-like nanostructures (see Figure 1a and Figure 1b) is very interesting and promising, thanks to high crystallinity that increases the electrical conductivity in two dimensions. Further studies are necessary to obtain substrates made only by nanowalls due to limitations related to the growth technique.

The voltage drop Δ*V* across the sample has been measured as a function of the applied temperature difference Δ*T*. Figure 1c shows the dependence of the temperature difference Δ*T* and the corresponding voltage Δ*V* measured for ZnO nanowire samples as a function of time. From our experimental data, the generated voltages can be plotted as a function of the applied temperature difference, as show in Figure 1d in order to estimate the Seebeck coefficient of the materials.

For all of the nanostructures obtained at different temperatures the Seebeck coefficient has been estimated by fitting the experimental data with a linear function as described in [23].

**Figure 2:**
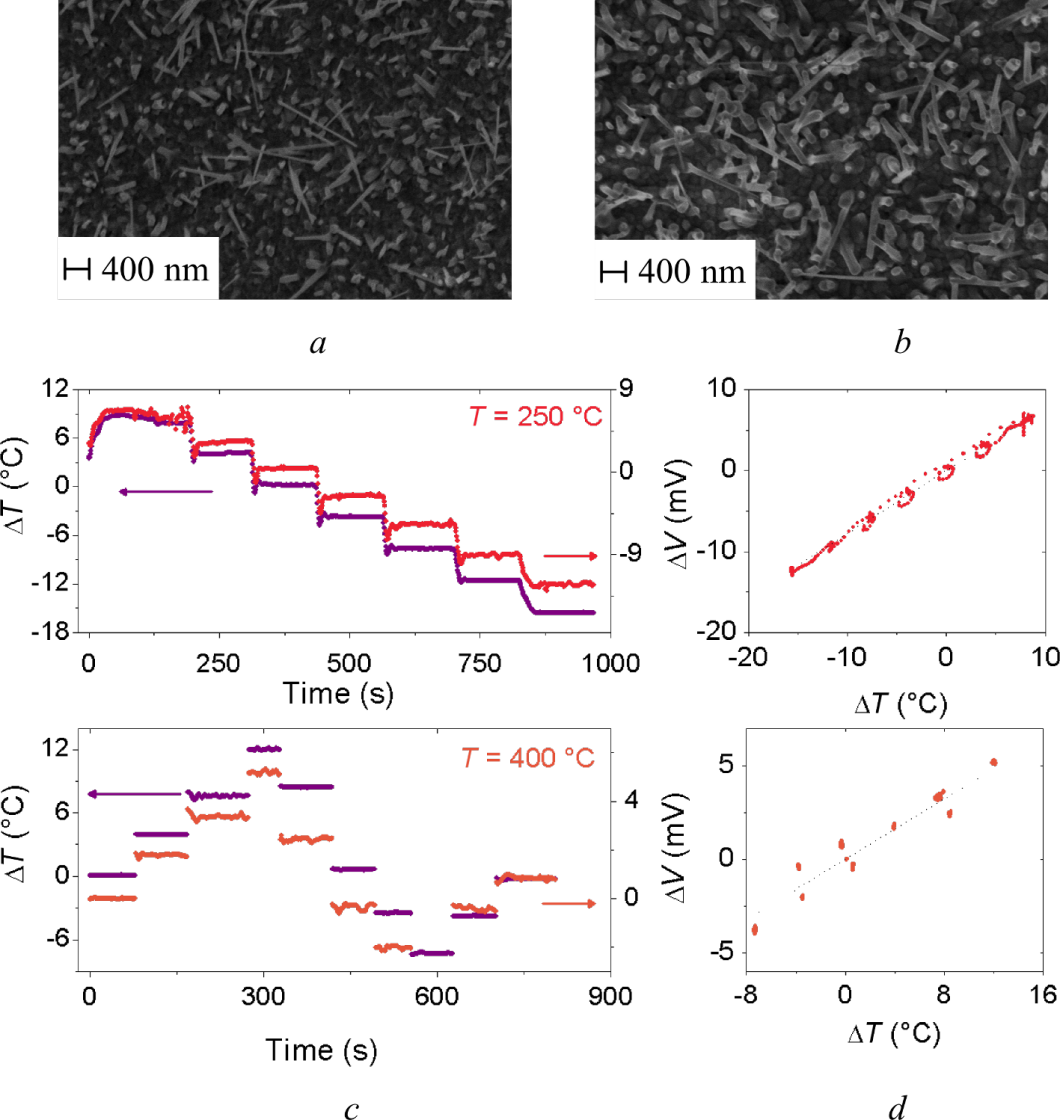
SEM images of CuO nanowires fabricated at (a) 250 °C and (b) 400 °C. (c) Dependence of the applied temperature difference Δ*T* and the thermoelectric voltage Δ*V* and (d) the generated voltage Δ*V* versus the applied temperature difference Δ*T* across the fabricated CuO samples.

Figure 2a and Figure 2b show the SEM images of nanostructured CuO synthesized via thermal oxidation at 250 °C and 400 °C, respectively. Similarly to ZnO, the deposition temperature had a strong influence on the morphology of the nanostructures. The diameter of CuO nanowires grown at 400 °C was almost doubled compared to CuO grown at 250 °C. Moreover, the nanowires showed a higher degree of vertical alignment compared to the ones fabricated at lower oxidizing temperature.

Figure 2c shows the applied temperature difference Δ*T* and the voltage Δ*V* for CuO nanowire samples. As reported in Figure 2d, the thermoelectric voltage Δ*V* and the applied temperature difference Δ*T* show a similar trend as expected for Seebeck effect for *p*-type materials as CuO.

The experimental values of the Seebeck coefficients of the ZnO and CuO nanowire samples are summarized in Table 1. The experimental results are in agreement with recently reported values of Seebeck coefficient for ZnO thin-films [25] and nanostructures [26–27] and CuO thin-film [28–29]. The sheet resistance *R*_s_ of the elements has been measured by a four-probe technique (Table 1).

**Table 1:** Measured Seebeck coefficients of the fabricated ZnO and CuO nanowires samples.

Material	Sheet resistance *R*_s_ [kΩ/sq]	Deposition temperature *T* [°C]	Seebeck coefficient α_m_ [mV/°C]

ZnO	100–300	700	−0.19
		870	−0.11
		1070	−0.10

CuO	<100	250	+0.82
		400	+0.43

Thermoelectric power factor (TPF) was estimated for both CuO and ZnO nanowires, based on sheet resistance *R*_s_. The electrical conductivity was calculated as σ = 1/(*R_s_*·*h*), where *h* is the thickness of each strip. We found values of σ of 2.0 S/m for copper oxide and 0.7 S/m for zinc oxide. While for CuO nanowires the electrical conductivity in consistent with literature [5], the electrical conductivity of ZnO nanowires is orders of magnitude lower than expected [30]. We believe that in the case of CuO nanowires, the bulk approximation for the estimate of electrical conductivity is appropriate, however this is not true for ZnO. In fact, ZnO nanowires mat is composed by long nanowires (>5 µm) and nanowalls, resulting in a very porous-like structure. The electrical conductivity is consequently underestimated using the bulk approximation, because of the density difference. Due to the strong influence of electrical conductivity in the expression of material thermoelectric power factor, it turns out that TPF_CuO_ is much higher than TPF_ZnO_, in particular 369.8 nW/(mK^2^) for CuO and 8.1 nW/(mK^2^) for ZnO nanowires. The performance of the prototype thermoelectric module will be thus somehow limited by the individual performances of ZnO nanostructures.

ZnO and CuO nanowires were assembled in a single device in order to fabricate a thermoelectric module. ZnO nanowires have been grown first; afterwards, copper oxide nanowires were synthesized on the same substrate, resulting in a series of five ZnO–CuO strips. In the planar thermoelectric device, ZnO TEG element has been grown at a deposition temperature of 870 °C, for which the largest conductivity and best nanowires coverage can be obtained. About CuO, although the Seebeck coefficient of the CuO oxidized at 400 °C is half the value of the one oxidized at 250 °C, the material treated at 400 °C was much more conductive, stable and completely oxidized compared to sample obtained at 250 °C. Thus, we have chosen 400 °C as oxidizing temperature for CuO nanowires.

**Figure 3:**
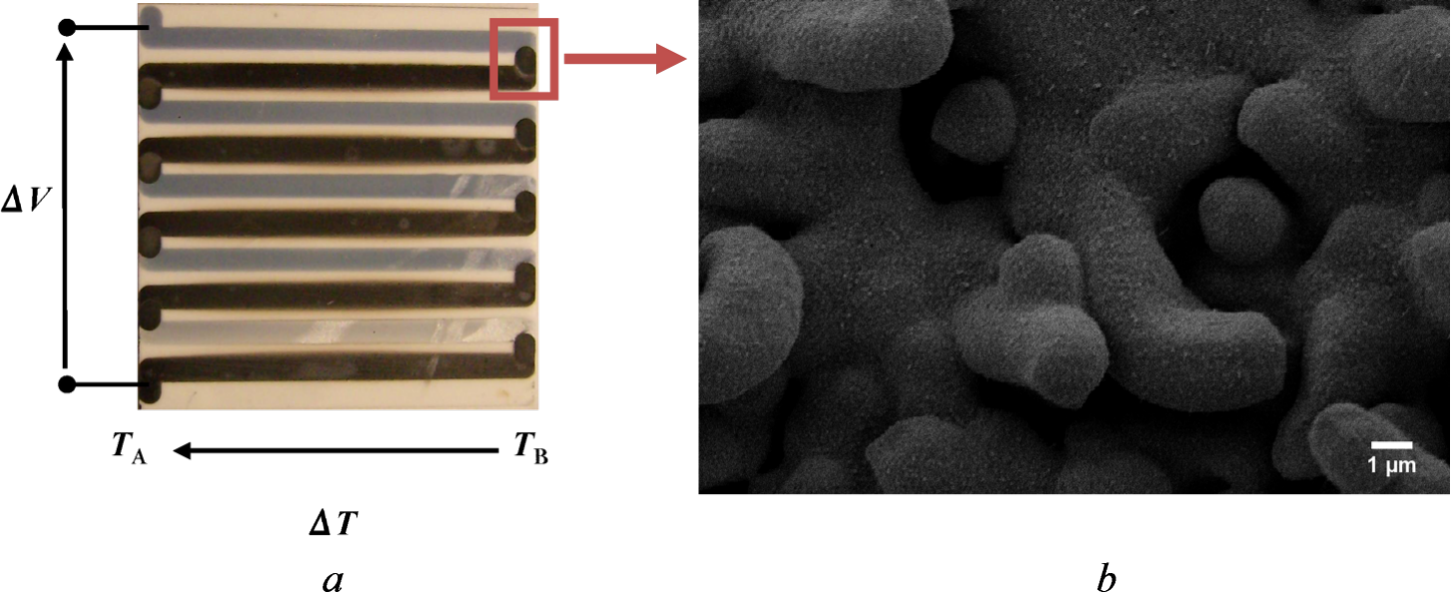
(a) Optical image of fabricated planar thermoelectric device based on ZnO and CuO nanowires. (b) SEM picture of the ZnO–CuO junction area.

Figure 3a shows a top-view optical image of the planar thermoelectric devices, fabricated combining ZnO and CuO nanowires-based multi-strips; the direction of applied temperature difference and thermoelectric voltage are also shown. Regarding the junction area between ZnO and CuO (Figure 3b), a metallic Cu film was deposited by sputtering on top of ZnO nanostructures and then oxidized in reactive atmosphere. ZnO nanowires were covered by CuO film/nanowires, thus forming a heterojunction.

**Figure 4:**
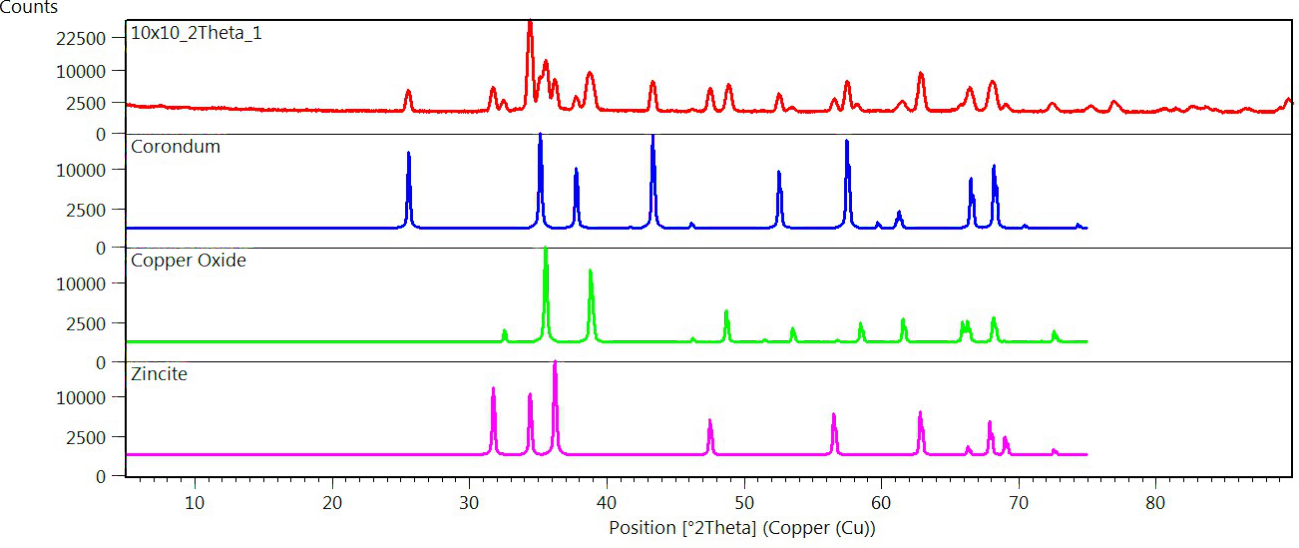
XRD spectra of the fabricated thermoelectric module (top to bottom as labeled).

X-ray diffraction has been carried out in order to investigate the structure of the thermoelectric module. We found evidence of the presence of three crystalline phases. Zincite (ZnO) and copper oxide (CuO) were found on corundum (Al_2_O_3_), the crystal structure of the alumina substrate (Figure 4) [31–32]. Figure 4 reports the spectra of the analytical reconstruction of each crystalline phase, and the total measured spectra in glancing-angle mode (ω = 1.5°), confirming the structure of the fabricated device.

**Figure 5:**
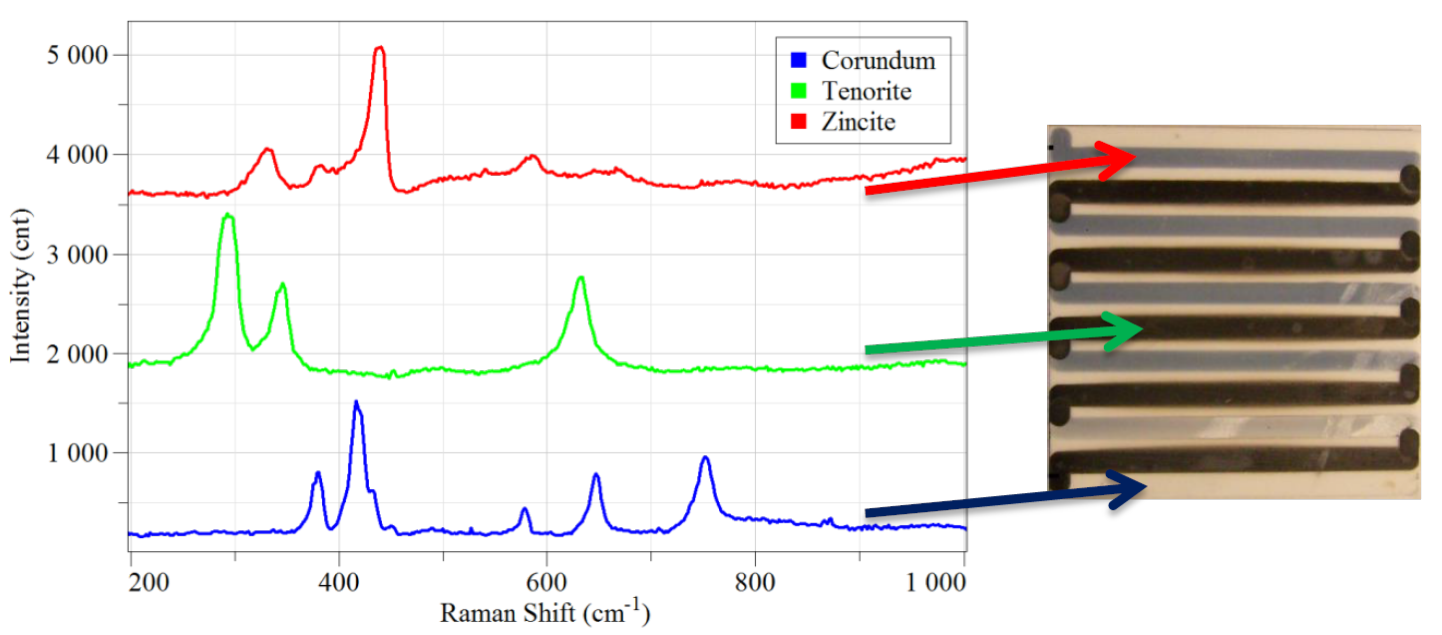
Raman spectra of the fabricated thermoelectric module for ZnO (red), CuO (green) and alumina substrate (in blue). Spectra shifted vertically and normalized for clarity purposes.

In addition, Raman spectroscopy has been carried out in order to verify the absence of contaminations between strips composed by different materials. Figure 5 reports the normalized Raman spectra of ZnO (zincite) and CuO (tenorite) strips, together with the spectrum of the bulk alumina (corundum) substrate. According to Raman spectra there is no overlapping in peaks position and no sign of cross contamination among the strips. Copper oxide exhibits three main peaks at 293, 344 and 631 cm^−1^, while zinc oxide peaks are located at 330, 380, 439 and 585 cm^−1^, respectively. The position of peaks agrees with values reported in literature [31–33]. Moreover, there is no sign of alumina substrate spectrum beneath the strips, as shown in Figure 5.

**Figure 6:**
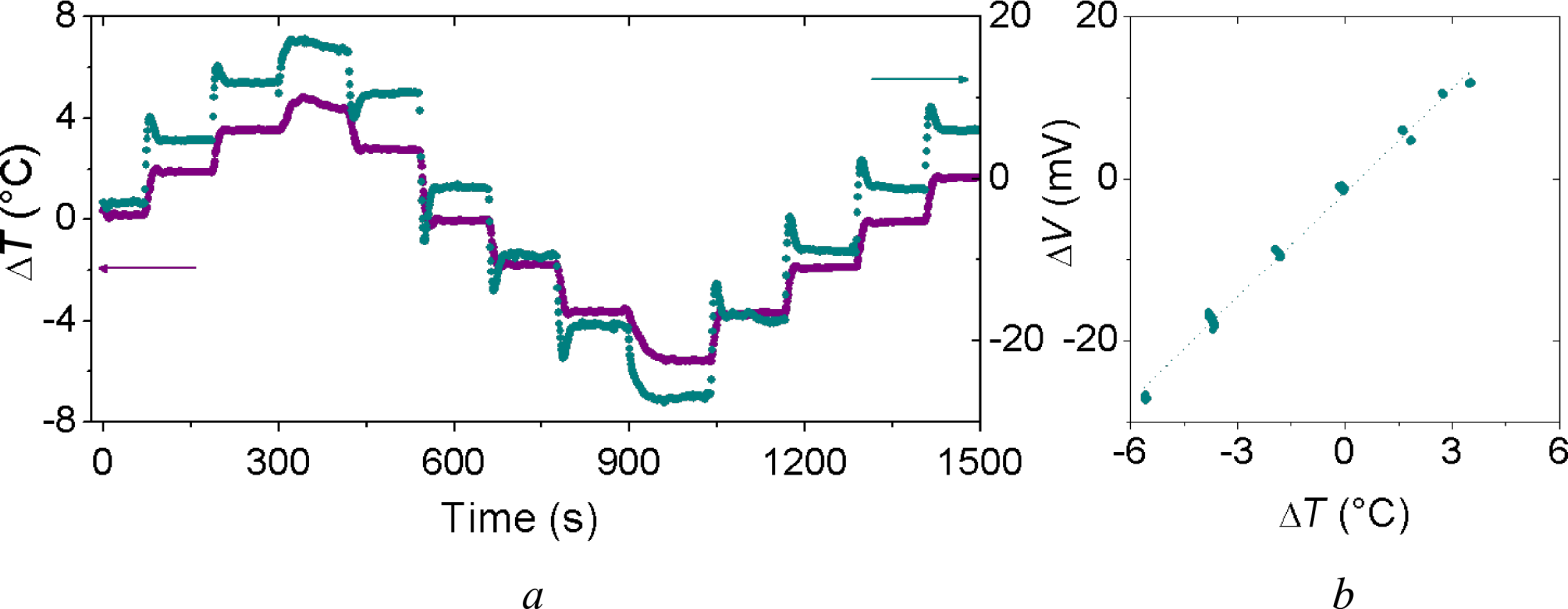
(a) Dependence of the applied temperature difference Δ*T* and the thermoelectric voltage Δ*V* as function of time and (b) the generated voltage Δ*V* versus the applied temperature difference Δ*T* for the planar thermoelectric device.

Figure 6a and Figure 6b show the measured voltage Δ*V* as a function of time with the applied temperature difference Δ*T*, and versus the applied temperature difference Δ*T,* respectively.

Each thermocouple, composed by the electrical series of two nanostructured ZnO and CuO strips, exhibits an overall Seebeck coefficient α_Cuo,ZnO_ of about 0.8 mV/°C. Moreover, the thermoelectric coefficient *S* of the entire planar device was measured, resulting in approximately 4 mV/°C, confirming the correct behavior of the fabricated prototype planar thermoelectric device. The electrical resistance of the entire thermoelectric planar generation resulted about 9 MΩ.

The overall performances of a thermoelectric generator can be evaluated by means of two parameters. The first one only takes into account energy conversion performance, and it is defined as *P*_max_/Δ*T*^2^. This parameter is the maximum electrical power generated in case of matched-load conditions, per unit-squared temperature difference applied on the device. The second one considers the physical dimensions of the generator also, or the miniaturization level, and is defined as:

[1]
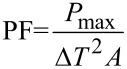


which is defined as the maximum power generated in matched-load conditions, respect to device area (*A*) and unit-squared temperature applied (Δ*T*^2^) [21]. It represents the matched-load power density, the maximum electrical power a generator could provide according to a defined area and temperature gradient.

The fabricated planar thermoelectric generators exhibits *P*_max_/Δ*T*^2^ of about 0.4 pW/K^2^, comparable with values reported in similar generators based on Si and Si-Ge nanowire arrays [34]. The power factor PF results of about 1 nW/K^2^m^2^: this value is low possibly due to the large dimensions of the fabricated TEG [2 cm × 2 cm].

## Conclusion

Quasi-monodimensional metal oxide nanowires are promising candidates for efficient thermoelectric energy conversion. ZnO (*n*-type) and CuO (*p*-type) nanowire bundles have been fabricated by physical processes and experimentally characterized, by means of morphological and structural investigations, evaluating the thermoelectric performance of the materials also. In particular, the Seebeck coefficient, the electrical conductivity and the TPF of the both materials were estimated around room temperature, in the range of 10–50 °C.

Combining both *n*- and *p*-type nanowire elements (ZnO and CuO, respectively), a prototype of planar thermoelectric module has been fabricated. The prototype device exhibited an overall thermoelectric Seebeck coefficient *S* of about 4 mV/°C and electric resistance of about 9 MΩ. The maximum electrical power on matched-load conditions generated by the entire thermoelectric device per unit-squared temperature was about 0.4 pW/K^2^ and the power density per unit-squared temperature resulted of about 1 nW/K^2^m^2^. As demonstrated by Xu et al., the performance of thermoelectric can be enhanced by improving electrical contact resistances; moreover, an increase in carrier concentration will boost the thermoelectric performance [34]. The main limitation of this prototype is to be found in the still high electrical resistance of the material, especially ZnO, limiting both the power factor and the figure of merit ZT. In particular, in order to evaluate ZT is necessary to measure the thermal conductivity of the materials. Thermal conductivity measurements are still ongoing, because we need the reengineering of the test device to get rid of the thermal influence of the substrate.

We believe that the presented thermoelectric generator can be further optimized, in particular with respect to the device size. Nevertheless, we have demonstrated the feasibility of the fabrication of planar thermoelectric generators based on mats of metal oxide nanowires. Optimization of the TPF of single thermoelectric materials (ZnO and CuO) and TEG geometrical dimension miniaturization is required to fabricate high-performance TEGs.

## Experimental

### Nanowires growth

As widely reported in literature [35], Zinc oxide is an intrinsic *n*-type semiconductor with a large direct band-gap of 3.3 eV, presenting a wurtzite crystalline structure. In order to fabricate zinc oxide nanowires, we used thermal evaporation process, which involves both vapour-phase (VP) and vapour-liquid-solid (VLS) growth mechanisms [36–37]. The deposition technique consists of the evaporation of bulk metal oxides powder followed by condensation of oxide vapour on the destination substrate. Due to a number of factors, including condensation temperature, pressure inside the alumina tube, inert carrier gas flux and process catalyst, it’s possible to promote the nucleation of the oxide powder on the substrate and thus synthesize metal oxide nanowires. The experimental set-up for the nanowires growth included a PC-controlled alumina tubular furnace (Lenton, UK) capable of reach high temperatures (≈1500 °C), which are indeed needed to initiate the decomposition of metal oxide and thus promoting its evaporation. The precision in the control of pressure, temperature gradient and gas carrier flux are key-factors for reproducibility of deposition and thus of samples. During the temperature transients (from room temperature to evaporation temperature and back to room temperature), inert gas was flown in reverse direction (from the substrate to the powder source) to avoid undesired condensation over the substrate. High-purity alumina substrates (20 mm × 20 mm, Kyocera, Japan) have been used as target substrate for both morphological and electrical investigations. Substrates have been cleaned in acetone using ultrasonic bath for 10 min and then dried with synthetic air. Gold nanoparticles have been deposited by RF magnetron sputtering (70 W Ar plasma for 5 s at room temperature, pressure 5 × 10^−3^ mbar) on the substrate, as they will act as catalyst for the nanowire growth. This technique was very easy and straightforward to use and allowed a good control of the density of nanoparticles. However was not possible to tune finely the size and the location of these nanoparticles. There are other techniques to decorate with a pattern the surface of the substrates, but they are much more expensive and not suitable for mass production. For example, it is possible to use focused ion beam (FIB) lithography to deposit actively each gold nanoparticles, but it is very time consuming and there is no demonstration of improved performances of the device. The ability to control the size and dispersion of the catalyst is a key parameter to control both the size and the density of nanowires on the substrate.

Both Au-sputtered substrates and ZnO powder (99% purity, Sigma-Aldrich Corporation, St. Louis, MO, USA) have been placed inside the tubular furnace, and the internal temperature (central region) has been set at 1370 °C; each substrate has been placed at lower temperatures (700 °C, 870 °C and 1070 °C) at different distances from the powder. Ar has been used as transport gas, with an internal pressure in the tube of 100 mbar. The deposition time, or the time of the direct flow of the transport gas from the powder to the substrates, has been set at 30 min [23].

Copper oxide is a *p*-type semiconductor with a narrow band-gap of 1.2 eV with monoclinic crystal structure [38]. In the present work, copper oxide nanowires have been grown by thermal oxidation of metallic Cu thin-film layer, previously deposited by RF sputtering on 20 mm × 20 mm alumina substrates [24].

Samples have been first cleaned in acetone using ultrasonic bath for 10 min and then dried with synthetic air. Then, a thin layer of metallic Cu has been deposited on samples by RF magnetron sputtering (50 W Ar plasma at room temperature, pressure 5 × 10^−3^ mbar, thickness 1 μm).

Samples have been placed in a quartz holder inside the alumina tubular furnace at a fixed temperature (250 °C and 400 °C), gas flow (300 sccm, 80% O_2_ and 20% Ar) and oxidation time (15 h) in order to oxidize the metallic layer and promote the growth of nanowires. The correct combination of temperature, atmosphere composition and oxidation time enables the nucleation due to mechanical stress, and the local oxidation of the metallic copper leads to the formation of nanowire on the surface of the film.

**Figure 7:**
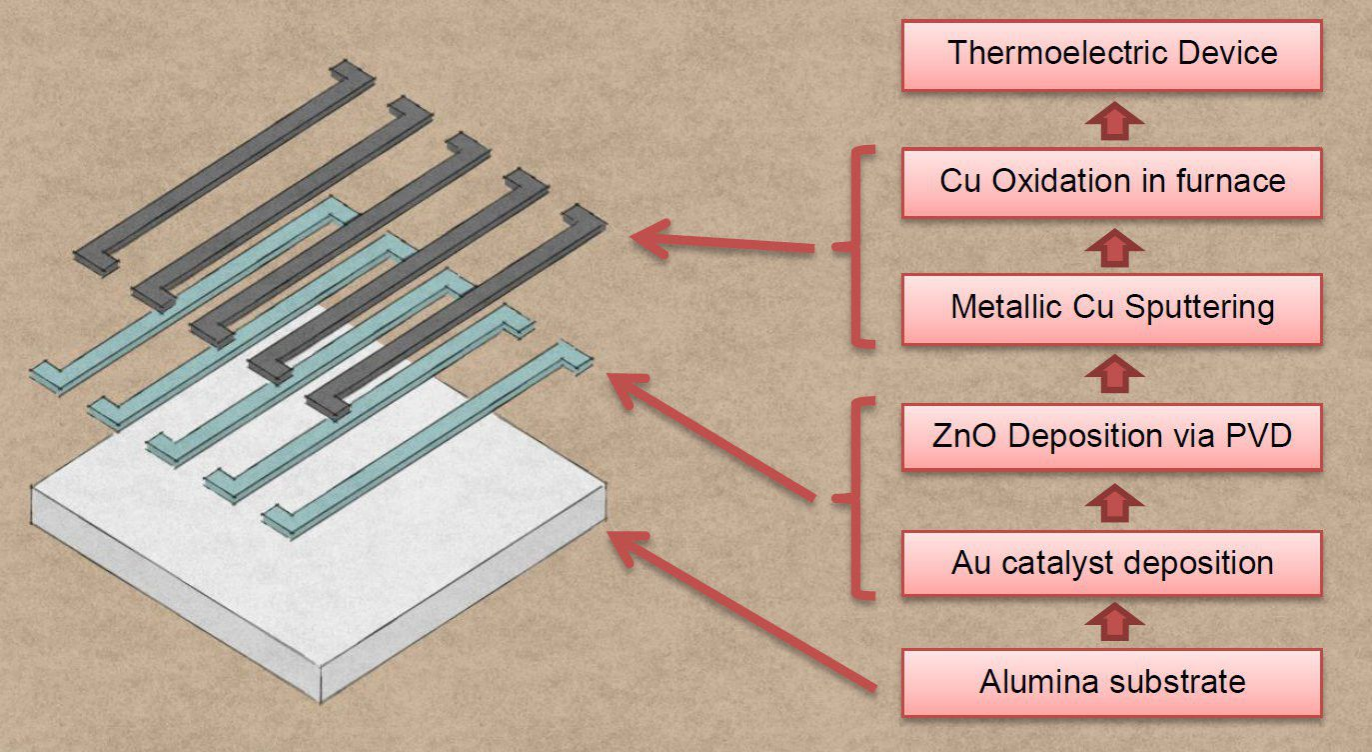
Schematics of the fabrication process.

In order to fabricate the prototype thermoelectric module, semiconducting materials have been deposited via patterned shadow mask technique on alumina substrates (20 mm × 20 mm, Kyocera, Japan) to form an array of elementary units. Each element consisted of an *S*-shaped strip 20 mm in length and 1 mm in width [22]. The electrical contact was provided by the overlap of adjacent strips, as reported in Figure 7.

ZnO nanowires have been grown by previously described thermal evaporation technique before copper deposition, to avoid undesired condensation of zinc oxide nanostructures on metallic copper. Au-catalysed samples were placed at 870 °C inside the furnace. The pressure inside the alumina tube was kept at 100 mbar, with an argon flow of 10 sccm (30 min deposition). Afterwards, copper oxide nanowires were synthesized by thermal oxidation. Copper metallic film was deposited via RF magnetron sputtering, as described before, using a 50 W argon plasma at room temperature (thickness 2 µm, 5 × 10^−3^ mbar pressure). Samples were then placed in the furnace and oxidized at 400 °C for 12 h in 80% oxygen/20% argon atmosphere (300 sccm flow).

### Morphological and structural characterization

The morphology of the nanowires has been investigated by Scanning Electron Microscopy (FE-SEM LEO 1525). Samples were attached to metallic stub using carbon glue. The electronic beam was operated in the range of 3–5 kV.

X-ray diffraction spectroscopy (XRD) was performed using an Empyrean diffractometer (PANalytical, Almelo, The Netherlands) mounting a Cu-LFF tube operated at 40 kV and 40 mA. XRD spectra were recorded by a parallel-plate collimated proportional Xe detector with a nickel large-β filter, in glancing-angle mode in the range of 5–90 degree (ω = 1.5°).

Raman characterization was performed by using a HORIBA (Kyoto, Japan) monochromator iHR320 configured with a grating of 1800 g/mm, coupled to a Peltier-cooled Synapse CCD. A He–Cd laser (442 nm) was focused on the samples by a fiber coupled confocal optical microscope (HORIBA) at 100× magnification. Spectra were recorded in the wavelength range 200–1000 cm^−1^.

### Experimental set-up for measurement of the thermoelectric response of metal oxide nanowires

**Figure 8:**
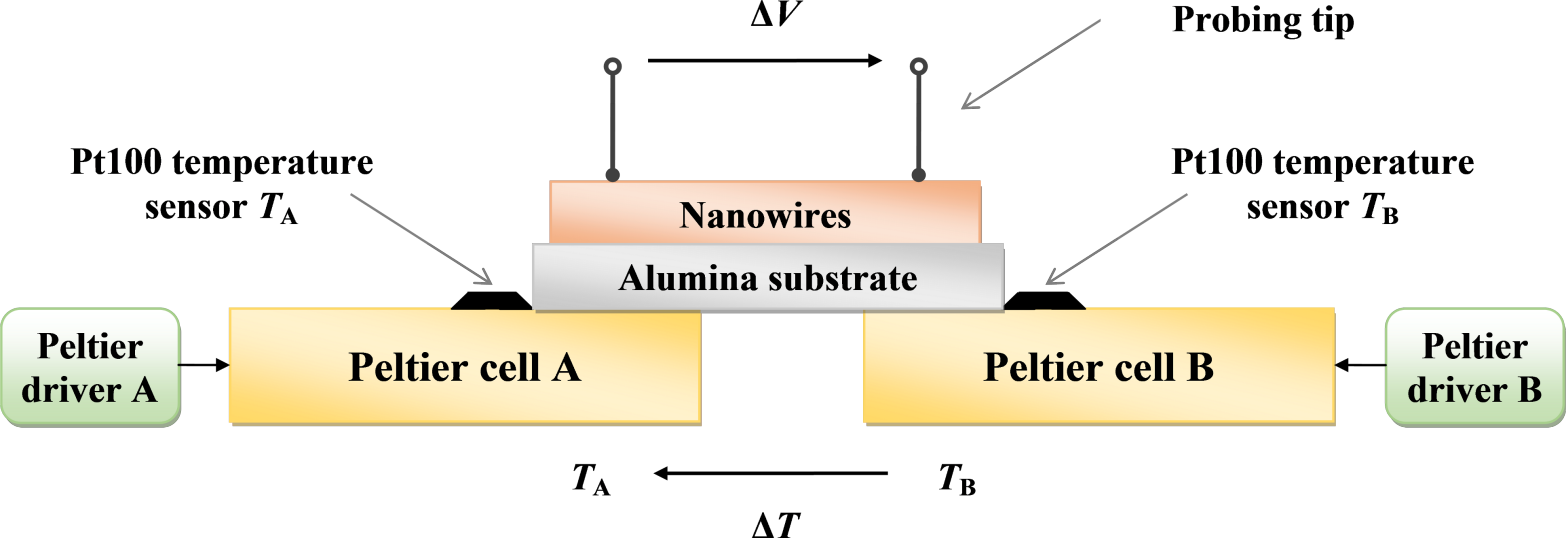
Schematic diagram (side view) of the experimental set-up for the measurement of the thermoelectric response of nanowires-based samples (not to scale).

The fabricated samples were experimentally characterized by measuring their thermoelectric response as a function of the applied temperature difference Δ*T* using a purposely developed experimental set-up based on two Peltier cells with relative driver stages and two reference Pt100 sensors and a PC-based control and acquisition system, as shown in Figure 8.

The edges of the samples were kept at the temperatures *T*_A_ and *T*_B_ by means of the Peltier cells and the associated controllers. Assuming that the temperatures *T*_A_ and *T*_B_ are uniform on each Peltier cell, the temperature difference Δ*T* applied to the sample has been numerically calculated from measured data *T*_A_ and *T*_B_ according to the relationship:

[2]
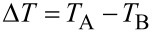


The measurements on the ZnO and CuO nanowire mats have been performed by means of a pair of probing tips, which connect the nanowire mat to an electronic measurement unit, forming a thermocouple made by the tested material and the probe material. The thermoelectric voltage Δ*V*, detected at the ends of the probing tips as a function of the temperature difference Δ*T*, provides a measurement of the relative Seebeck coefficient of the metal oxide nanowire mat with respect to the probe material. As reference thermoelectric material for the probing tips, Chromel was used. Chromel is an alloy of nickel and chromium, which exhibits a positive absolute Seebeck coefficient of about 28.1 μV/K [39] and is commonly used with Alumel to form *K*-type thermocouples.

The voltage Δ*V*, measured at the ends of the probing tips, is proportional to the applied temperature difference Δ*T* as follows:

[3]
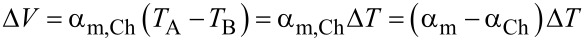


where α_m,Ch_ is the Seebeck coefficient of the tested material (ZnO or CuO) labeled m, with respect to the reference material Ch, i.e. Chromel; while α_m_ and α_Ch_ are the absolute Seebeck coefficients of the tested material m and the Chromel, respectively. By convention, the thermoelectric voltage Δ*V* represents the potential of the cold side with respect to the hot side [39], as shown in Figure 8.

For the characterization of the planar thermoelectric device, the voltage Δ*V* generated by the ZnO–CuO thermocouples has been measured using a pair of copper probing tips by means of the above-described experimental set-up, as a function of the applied temperature difference Δ*T*. The thermoelectric voltage Δ*V* is proportional to the applied temperature difference Δ*T* according to the following equation:

[4]
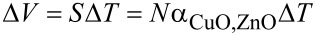


where *S* and α_Cuo,ZnO_ are, respectively, the Seebeck coefficient of the entire thermoelectric device and of a single ZnO–CuO thermocouple and *N* = 5 the number of the elements which composes the thermoelectric device.

The voltage Δ*V* has been amplified by means of a low-noise instrumentation amplifier INA111 with a gain of 100 for the characterization of both metal oxide nanowires and planar thermoelectric device.

## References

[1] Karni J (2011). Nat Mater.

[2] Kraemer D, Poudel B, Feng H-P, Caylor J C, Yu B, Yan X, Ma Y, Wang X, Wang D, Muto A (2011). Nat Mater.

[3] Nolas G S, Sharp J, Goldsmid H J (2001). Thermoelectrics: basic principles and new materials developments.

[4] Pichanusakorn P, Bandaru P (2010). Mater Sci Eng, R.

[5] Walia S, Balendhran S, Nili H, Zhuiykov S, Rosengarten G, Wang Q H, Bhaskaran M, Sriram S, Strano M S, Kalantar-zadeh K (2013). Prog Mater Sci.

[6] O'Dwyer M F, Humphrey T E, Linke H (2006). Nanotechnology.

[7] Lee C-H, Yi G-C, Zuev Y M, Kim P (2009). Appl Phys Lett.

[8] Vomiero A, Concina I, Comini E, Soldano C, Ferroni M, Faglia G, Sberveglieri G (2012). Nano Energy.

[9] Tsubota T, Ohtaki M, Eguchi K, Arai H (1997). J Mater Chem.

[10] Wang W, Jia F, Huang Q, Zhang J (2005). Microelectron Eng.

[11] Aksamija Z, Knezevic I (2010). J Comput Electron.

[12] Vineis C J, Shakouri A, Majumdar A, Kanatzidis M G (2010). Adv Mater.

[13] Stranz A, Sökmen Ü, Kähler J, Waag A, Peiner E (2011). Sens Actuators, A.

[14] Lieberman A, Leanna A, McAlonan M, Heshmatpour B (2007). AIP Conf Proc.

[15] Determan W R, Otting W, Frye P, Abelson R, Ewell R, Miyake B, Synder J (2007). AIP Conf Proc.

[16] Yang J, Caillat T (2006). MRS Bull.

[17] Yang J (2005). Potential applications of thermoelectric waste heat recovery in the automotive industry. ICT 2005. 24th International Conference on Thermoelectrics 2005.

[18] Yu C, Chau K T (2009). Energy Convers Manage.

[19] Rowe D M, Min G (1998). J Power Sources.

[20] Beeby S, White N M (2010). Energy Harvesting for Autonomous Systems.

[21] Dalola S, Ferrari M, Ferrari V, Guizzetti M, Marioli D, Taroni A (2009). IEEE Trans Instrum Meas.

[22] Dalola S, Faglia G, Comini E, Ferroni M, Soldano C, Zappa D, Ferrari V, Sberveglieri G (2012). Procedia Eng.

[23] Dalola S, Faglia G, Comini E, Ferroni M, Soldano C, Zappa D, Ferrari V, Sberveglieri G (2011). Procedia Eng.

[24] Zappa D, Comini E, Zamani R, Arbiol J, Morante J R, Sberveglieri G (2013). Sens Actuators, B.

[25] Cheng H, Xu X J, Hng H H, Ma J (2009). Ceram Int.

[26] Kinemuchi Y, Mikami M, Kobayashi K, Watari K, Hotta Y (2010). J Electron Mater.

[27] Fang L, Yang X F, Peng L P, Zhou K, Wu F, Huang Q L, Kong C Y (2010). J Supercond Novel Magn.

[28] Muhibbullah M, Hakim M O, Choudhury M G M (2003). Thin Solid Films.

[29] Chang H, Kao M-J, Cho K-C, Chen S-L, Chu K-H, Chen C-C (2011). Curr Appl Phys.

[30] Han J, Mantas P Q, Senos A M R (2001). J Eur Ceram Soc.

[31] Luo M-F, Fang P, He M, Xie Y-L (2005). J Mol Catal A: Chem.

[32] Zappa D, Comini E, Sberveglieri G (2013). Nanotechnology.

[33] Alim K A, Fonoberov V A, Shamsa M, Balandin A A (2005). J Appl Phys.

[34] Xu B, Li C, Myronov M, Fobelets K (2013). Solid-State Electron.

[35] Janotti A, Van de Walle C G (2009). Rep Prog Phys.

[36] Comini E (2006). Anal Chim Acta.

[37] Soldano C, Comini E, Baratto C, Ferroni M, Faglia G, Sberveglieri G (2012). J Am Ceram Soc.

[38] Kaur M, Muthe K P, Despande S K, Choudhury S, Singh J B, Verma N, Gupta S K, Yakhmi J V (2006). J Cryst Growth.

[39] Kasap S O (2006). Principles of electronic materials and devices.

